# Decomposition of gendered income-related inequalities in multiple biological cardiovascular risk factors in a middle-aged population

**DOI:** 10.1186/s12939-018-0804-2

**Published:** 2018-07-13

**Authors:** Paola A. Mosquera, Miguel San Sebastian, Anneli Ivarsson, Per E. Gustafsson

**Affiliations:** Epidemiology and Global Health, Department of Public Health and Clinical Medicine, Umeå University, SE-901 87 Umeå, Sweden

**Keywords:** Income inequality, Cardiovascular risk factors, Middle age, Decomposition analysis, Sweden

## Abstract

**Background:**

Socioeconomic inequalities in cardiovascular disease seem to widen or endure in Sweden. However, research on inequalities in antecedent cardiovascular risk factors (CVRFs), and particularly what underpins them, is scarce. The present study aimed 1) to estimate income-related inequalities in eight biological cardiovascular risk factors in Swedish middle-aged women and men; and 2) to examine the contribution of demographic, socioeconomic, behavioural and psychosocial determinants to the observed inequalities.

**Methods:**

Participants (*N* = 12,481) comprised all 40- and 50-years old women and men who participated in the regional Västerbotten Intervention Programme in Northern Sweden during 2008, 2009 and 2010. All participants completed a questionnaire on behavioural and psychosocial conditions, and underwent measurements with respect to eight CVRFs (body mass index; waist circumference; total cholesterol; high-density lipoprotein cholesterol; low-density lipoprotein cholesterol; triglycerides; systolic/diastolic blood pressure; glucose tolerance). Data on cardiovascular risk, psychosocial and health behaviours were linked to national register data on income and other socioeconomic and demographic factors. To estimate income inequalities in each CVRF concentration indexes were calculated, and to examine the contribution of the underlying determinants to the observed inequalities a Wagstaff-type decomposition analysis was performed separately for women and men.

**Results:**

Health inequalities ranged from small to substantial with generally greater magnitude in women. The highest inequalities among women were seen in BMI, triglycerides and HDL-cholesterol (Concentration index = − 0.1850; − 0.1683 and − 0.1479 respectively). Among men the largest inequalities were seen in glucose regulation, BMI and abdominal obesity (Concentration index = − 0.1661; − 0.1259 and − 0.1172). The main explanatory factors were, for both women and men socioeconomic conditions (contributions ranging from 54.8 to 76.7% in women and 34.0–72.6% in men) and health behaviours (contributions ranging from 6.9 to 20.5% in women and 9.2 to 26.9% in men). However, the patterns of specific dominant explanatory factors differed between CVRFs and genders.

**Conclusion:**

Taken together, the results suggest that the magnitude of income-related inequalities in CVRFs and their determinants differ importantly between the risk factors and genders, a variation that should be taken into consideration in population interventions aiming to prevent inequalities in manifest cardiovascular disease.

## Background

Despite decreasing cardiovascular disease (CVD) morbidity and mortality rates over the last two decades, Sweden has seen a worrying development with widening socioeconomic inequalities in cardiovascular morbidity, mortality as well as in life expectancy, markedly patterned by education [1] and especially among women [2]. Whereas inequalities in manifest cardiovascular disease, which may appear and accentuate with aging [3] would be expected to be preceded by corresponding inequalities in cardiovascular risk factors (CVRFs), research on multiple CVRFs inequalities, and particularly what explains them, is scarce. The present study seeks to contribute to this topic by estimating income-related inequalities in a range of biological cardiovascular risk factors in middle-aged women and men, and decomposing them by demographic, socioeconomic, family, psychosocial and behavioural factors.

To understand the relative role that inequalities in different CVRFs play in shaping the demonstrated inequalities in manifest CVD [4], comprehensive investigations of multiple CVRFs are especially valuable as they give a more complete picture of precursor inequalities. Indeed, research has demonstrated persisting and clear inverse social gradients across a range of cardiovascular risk factors, such as glucose, blood pressure, blood lipids and obesity [5–10]. In Northern Sweden, increasing and decreasing trends have been observed for different CVRFs [11–14], with educational inequalities persisting in both CVRFs [11–14] and in manifest CVD [15]. However, despite increasing income inequalities in Sweden [16], most Swedish research has focused on education rather than income inequalities.

Since there are large gender differences in both cardiovascular health and socioeconomic conditions (such as income and education), it is reasonable to suspect that inequalities may differ between women and men. With a few exceptions [17, 18], most studies [5, 7, 10, 19–21], including Northern Swedish populations [11–14], have indeed found larger socioeconomic and educational inequalities in women, which contrasts to the generally worse cardiovascular profile in men during middle age. This illustrates the need to consider not only population averages but also the social and gendered population patterns of health and disease to develop appropriate interventions with an equity lens perspective [22].

In an effort to move beyond simple demonstrations of health inequalities and towards understanding their underpinnings, public health research has begun to incorporate measures such as the concentration index and decomposition analysis. In contrast to conventional regression models which are dealing with health outcomes, decomposition analysis is dealing with an outcome that summarize the population level health and its accumulation along the income distribution estimated by a concentration index. As such, the decomposition analysis is able to estimate the independent contributions of different factors to a concentration index, which is directly addressing the question of which factors explain a given health inequality [23].

The few studies that have decomposed socioeconomic inequalities in CVRFs have only studied single factors. Most of them have only addressed inequalities in obesity [17, 19–21] or behavioural factors [24, 25], and overall suggest that socioeconomic position, education, and health behaviours are the most common factors contributing to inequalities in both women and men [17, 19–21, 24]. Gender differences in the explanatory role of different factors have also been identified, where income and socioeconomic position seem to be more important for women [19, 20], while educational attainment may be more important for men [17, 24]. Demographics and family factors have been of lesser importance and differed among genders showing both positive and negative contributions to explain inequalities [17, 19, 21]. We have also recently reported that the early life roots of income inequalities in metabolic syndrome seem to differ between women and men [26]. However, the scarcity of studies simultaneously analysing the underlying factors for the inequalities and using comprehensive approaches including multiple CVRFs make it difficult to draw any conclusions about the determinants explaining inequalities in CVRFs in women and men.

To fill in these knowledge gaps on socioeconomic inequalities in CVRFs and their determinants, the present study aims 1) to estimate income-related inequalities in eight biological cardiovascular risk factors in Swedish middle-aged women and men; and 2) to examine the contribution of demographic, socioeconomic, behavioural and psychosocial determinants to the observed inequalities.

## Methods

### Population and data

The study used data from a county-wide preventive programme “Västerbotten Intervention Program” (VIP), implemented since 1990 in the county of Västerbotten in the northern part of Sweden. The present study included all VIP participants (*N* = 12,481) aged 40- and 50-years old in 2008, 2009 and 2010 [26], thus focusing on a comparatively young population before manifest CVD has become prevalent but when its precursors in CVRFs are common.

The VIP programme invites all individuals aged 40, 50, and 60 years (yrs) who live in the county to participate in a health examination at their local health care centres. During the health examination a number of cardiovascular risk markers are measured (e.g. Body Mass Index (BMI), blood pressure, glucose tolerance, blood lipids (Low-density and High-density lipoproteins (LDL and HDL)) and each individual completes a comprehensive questionnaire including questions related to self-reported health, lifestyle behaviours, social network and support, working conditions, physical activities, tobacco and alcohol consumption [27]. All participants also receive information about their results in an individual health dialogue with a trained nurse, where the relation between concomitant CVRFs and lifestyle habits are discussed, aiming to motivate and promote healthier lifestyles or other changes in the individual’s conditions. The design of the VIP programme, the content areas of the questionnaire as well as the response rates of the programme have been described in detail elsewhere [27, 28].

Cardiovascular risk, psychosocial and health behaviour data from VIP were linked to Swedish population register data through the Umeå SIMSAM Lab microdata infrastructure [29]. Demographic and socioeconomic data included in the Lab originate from the registers of Statistics Sweden (e.g. Integrated Database for Labour Market Research).

### Variables

#### Outcome measures

The outcomes of interest were cardiovascular risk factors measured during health examinations: BMI, waist circumference, HDL, LDL, triglycerides, total cholesterol, glucose (fasting and 2 h glucose tolerance test) and blood pressure. For details of the measures, see previous VIP reports [27]. In addition to the biological measurements, auxiliary information about self-reported antihypertensive and lipid medication and of diabetes diagnosis was used in order to avoid underestimation of the outcomes. To be able to utilize this auxiliary information, all outcomes were dichotomized following international classification guidelines on cardiovascular disease. See Table 1 for the specific outcomes operationalization.

**Table 1:** Outcome variable operationalization

Risk indicator	Lower risk (=0)	Higher risk (=1)	Reference
BMI (Body mass index)	< 30 kg/m^2^ (Normal, underweight and overweight)	≥ 30 kg/m^2^ (Obesity, severe/extreme obesity)	[51]
Abdominal obesity	Waist circumference Men: < 102 cm Women: < 88 cm (Normal or increased risk)	Waist circumference Men: ≥ 102 cm Women: ≥ 88 cm (Substantially increased risk)	[51]
LDL levels^a^ (Low-density Lipoprotein cholesterol)	≤ 4.9 mmol/L (Desirable to high)	> 4.9 mmol/L OR taking lipid treatment (Very High)	[52]
HDL levels (High-Density Lipoprotein cholesterol)	Men: ≥1.0 mmol/L Women:: ≥1.2 mmol/L (Normal risk)	Men: < 1.0 mmol/L Women: < 1.2 mmol/L (Increased risk)	[42]
Triglycerides	< 1.7 mmol/L (Normal risk)	≥1.7 mmol/L (Increased risk)	[42]
Total cholesterol^a^	< 6.5 mmol/L (Ideal to high)	≥ 6.5 mmol/L OR taking lipid treatment (Very or extremely high)	[42, 53]
Glucose regulation	Fasting capillary plasma glucose ≤6.0 mmol/L and 2 h plasma glucose ≤8.8 mmol/L (Normal)	Impaired fasting glucose (IFG): fasting capillary plasma glucose 6.1–6.9 mmol/L Impaired glucose tolerance (IGT): fasting plasma glucose < 7.0 and 2 h plasma glucose 8.9–12.1 mmol/L Diabetes: fasting plasma glucose ≥7.0 or/and 2 h plasma glucose ≥12.2 mmol/L OR self-reported diabetes (Hyperglycemia)	[54]
Blood pressure	< 140/90 mmHg (Optimal to Pre-hypertensive)	≥ 140/90 mmHg OR self-reported anti-hypertensive drug (Hypertensive)	[55]

^a^Cutt-off points for dichotomization were stablished at “very/extremely high” to identify those at highest risk and due to high prevalence of high total-cholesterol and LDL-cholesterol levels in the study population. The VIP programme applies cut-offs at lower levels for some variables and takes multiple risk factors into account to promote preventive activities at earlier stages

#### Socioeconomic indicator

The total earned income measured in the year of participation (2008, 2009 or 2010) was the variable used to capture the socioeconomic status and living standards. Total earned income includes all taxable earnings of an individual over the course of any given year, including income from employment, business if the person is self-employed, pension if the person is retired, long-term disability benefits received prior to minimum retirement age, and other taxable transfers such as parental leave benefits and unemployment benefits. It does not include income from capital, such as profit from renting or selling property, or other financial investments.

#### Determinants of inequalities

Variables considered as determinants of inequality in CVRFs included factors with known or plausible links to both cardiovascular disease or risk factors on the one hand, and to individual financial conditions on the other [30]: *Demographic variables* (age, year of participation); *socioeconomic conditions* (income quintiles, education, occupation and immigration status); *family conditions* (civil status, having children in the household); *geographical area* (inland/coastal with or without hospital); *health behaviours* (physical activity, tobacco and alcohol consumption); and *psychosocial factors* (availability of social interaction, availability of attachment and job strain) were included. All variables were categorical, coded as follows:

*Age* was categorized into two groups: 40 yrs. (1), 50 yrs. (2).

*Year of participation* was categorized into three groups: 2008 (1), 2009 (2), 2010 (3).

*Education* was categorized into seven groups according to the official standard Swedish educational classification [31]: Compulsory education less than 9 yrs. (1), compulsory education 9 yrs. (2), secondary education up to 2 yrs. (3), secondary education 3 yrs. (4), post-secondary education less than 3 yrs. (5), post-secondary education 3 yrs. or more (6) and postgraduate (7). Whereas the distribution of education across all seven levels are reported for descriptive purposes (Table 2), for the main analysis, levels 1 and 2 as well as 6 and 7 were collapsed due to the small sample size of levels 1 and 7.

**Table 2:** Description of characteristics (N, prevalence (%), and concentration index C) of VIP participants (*N* = 12,481) aged 40- and 50-years old in 2008–2010 by gender and income inequalities for each factor

	Women			Men		
	N	%	C	N	%	C
BMI						
Obesity, severe and extreme obesity	1160	18.1	−0.185*	1221	20.4	−0.126*
Abdominal obesity						
Substantially increased risk	2571	40.1	−0.142*	1804	30.1	−0.117*
HDL levels						
Low HDL	1055	25.1	−0.148*	785	19.2	−0.083*
LDL levels						
Very high LDL	297	4.7	−0.141*	579	9.9	−0.027
Triglycerides (TG)						
High TG	880	13.7	−0.168*	2002	33.4	−0.085*
Cholesterol						
Hypercholesterolemia	518	8.1	−0.077*	848	14.1	−0.033
Hyperglycemia						
IGT/IFG/Diabetes	952	15.2	−0.088*	909	15.3	−0.166*
Blood pressure						
Hypertensive	662	10.3	−0.002	1086	18.1	−0.053*
Age						
40 yrs	3115	48.2	−0.107*	2939	48.8	−0.026*
50 yrs	3344	51.8	0.110*	3083	51.2	0.024*
Year of participation						
2008	2205	34.1	−0.058*	2000	33.2	−0.040*
2009	2069	32.0	−0.013*	1939	32.2	−0.023*
2010	2185	33.8	0.070*	2083	34.6	0.063*
Total earned income						
Lowest quintile	127,974 kr	20.0	−1.000*	158,978 kr	20.0	−1.000*
2	218,018 kr	20.0	−0.501*	276,985 kr	20.0	−0.499*
3	258,629 kr	20.0	0.002	321,312 kr	20.0	−0.001
4	296,587 kr	20.0	0.502*	374,109 kr	20.0	0.500*
Highest quintile	409,312 kr	20.0	1.000*	531,111 kr	20.0	1.000*
Education level						
Compulsory education less than 9 yrs	60	0.9	−0.602	41	0.7	− 0.627*
Compulsory education 9 yrs	298	4.6	−0.386*	447	7.4	−0.247*
Secondary education up to 2 yrs	2163	33.6	−0.260*	2856	47.5	−0.158*
Secondary education 3 yrs	967	15.0	−0.095*	656	10.9	−0.048*
Post-secondary education less than 3 yrs	1156	17.9	0.115*	924	15.4	0.172*
Post-secondary education 3 yrs. or more	1676	26.0	0.332*	930	15.5	0.215*
Postgraduate	123	1.9	0.647*	155	2.6	0.567*
Occupation						
Managers	285	4.6	0.662*	515	9.0	0.455*
Upper professionals	1243	20.2	0.458*	931	16.2	0.309*
Middle non-manual	1284	20.9	0.150*	999	17.4	0.253*
Lower non-manual	679	11.0	−0.161*	249	4.3	−0.189*
Skilled manual	2292	37.3	−0.329*	2808	48.9	−0.345*
Unskilled manual	366	6.0	−0.556*	243	4.2	−0.455*
Immigrant status	623	9.7	−0.216*	492	8.2	−0.227*
Civil status						
Unmarried	2038	31.6	−0.073*	2444	40.6	−0.176*
Married	3587	55.5	0.049*	3004	49.9	0.193*
Divorced	773	12.0	0.018	563	9.4	−0.082*
Widowed	61	0.9	0.175*	11	0.2	0.428*
Children in the household	3759	58.2	−0.030*	3369	56.0	0.135*
Geographical area						
Coastal with hospital	4582	70.9	0.168*	4214	70.0	0.195*
Coastal without hospital	852	13.2	−0.135*	785	13.0	−0.153*
Inland with hospital	382	5.9	−0.038	337	5.6	−0.047
Inland without hospital	620	9.6	−0.189*	656	10.9	−0.213*
Other	23	0.4	−0.227*	30	0.5	−0.154
Physical activity						
Sedentary	1147	17.8	−0.130*	1616	26.8	−0.128*
Moderate activity	3040	47.1	−0.070*	2638	43.8	−0.041*
Physically active	2272	35.2	0.160*	1768	29.4	0.170*
Smoking						
Never smoked	3693	57.7	0.051*	3804	64.1	0.145*
Former smokers	1873	29.3	0.008	1383	23.3	−0.066*
Current smokers	837	13.1	−0.117*	747	12.6	−0.193*
Snus use						
Never used	4928	77.6	−0.008	3120	52.5	0.007
Former users	648	10.2	0.045*	1194	20.1	0.040*
Current users	774	12.2	−0.021	1632	27.5	−0.042*
Alcohol disorder test						
Not at risk	5569	93.2	−0.065*	4952	87.6	0.063*
Probably risk alcohol consumption	386	6.5	0.065*	641	11.3	−0.037
Probably alcohol dependence	22	0.4	0.038	61	1.1	−0.265*
Availability of social interaction						
Low	2024	31.3		1949	32.4	
Extended	4435	68.7	0.201*	4073	67.6	0.135*
Availability of attachment						
Low	1671	25.9		2613	43.4	
Strong	4788	74.1	0.106*	3409	56.6	0.042*
Job strain						
Low strain	1057	16.4	−0.013	1194	19.8	−0.022
Passive	1476	22.9	−0.280*	1338	22.2	−0.222*
Active	2159	33.4	0.290*	2206	36.6	0.251*
High strain	1767	27.4	−0.068*	1284	21.3	−0.100*

%: prevalence (column percentage) of each category within each sex. C: Concentration index. * indicates that C differs from 0; *p* < 0.05. *BMI* Body Mass Index, *LDL* Low-Density Lipoprotein Cholesterol, *HDL* High-Density Lipoprotein Cholesterol, *TG* Triglycerides, *IGT* Impaired glucose tolerance, *IFG* Impaired fasting glucose

*Immigrant status* was defined as immigrant (1) if the individual at some time after birth has migrated to Sweden, and non-immigrant (0) otherwise.

*Occupation* was categorized into 6 groups [32]: (1) Managers, (2) upper professionals, (3) middle non-manual workers, (4) lower non-manual workers, (5) skilled manual workers, (6) unskilled manual workers. For the main analyses, groups 1 and 2 were collapsed due to the small sample size of group 1.

*Civil status* was categorized into four groups: unmarried (1), married (2), divorced/separated (3), and widowed (4).

*Having children in the household* was defined as couple/co-habiting/single without child (0) and couple/co-habiting/single with child 0–18 yrs. living at home (1).

*Geographical area* was based on the Västerbotten municipality in which each participant was registered, and categorized into: coastal municipality with hospital (1), coastal municipality without hospital (2), inland municipality with hospital (3), inland municipality without hospital (5), and municipality outside Västerbotten (5).

*Physical activity* was measured through three items that explore commuting activity, leisure activities and frequency of physical exercise. In concordance with previous studies conducted on the VIP population [33] the items were combined and individuals were categorized into three groups: Sedentary (1), moderately active (2), and physically active (3).

*Tobacco habits* are based on two different questions. Guided by previous studies conducted on the VIP population [34] *Smoking* was categorized into three groups: Never smoked (1), former smokers (2) and current daily or intermittent smokers (3). *Use of Swedish moist snuff (snus use)* was categorized into three groups: Never used (1), former users (2) and current users (3).

*Alcohol consumption* was measured through the items from “The Alcohol Use Disorder Identification Test” (AUDIT-questionnaire [35]). This variable was categorized according to AUDIT scores as: Not at risk: < 8 points for men and <  6 for women (1), Hazardous/harmful alcohol consumption: 8–15 points in men, 6–13 points in women (2), and Alcohol abuse/dependence: ≥ 16 points in men, ≥14 points in women (3).

*Social support* including social network (availability of social integration (AVSI)) and emotional support (availability of attachment (AVAT)), was measured through an abbreviated version of the Interview Schedule for Social Interaction [36]. Consistent with previous studies conducted on the VIP population [37], emotional support and social network items were added up and then dichotomized into high (1) and low (0) AVAT and AVSI by the mean value.

*Job strain* was measured through the items from the Karasek demand-control questionnaire [38]. As in previous studies conducted on the VIP population [37], categories were defined according to the Karasek demand-control model. Psychological demands and decision latitude items were added up and then dichotomized by the median score. The cross-classification of job demands and job control according to their gender-specific medians produced four categories: low strain = low demands + high control (1), passive = low demands + low control (2), active = high demands + high control (3), and high strain = high demands + low control (4).

### Statistical analysis

#### Drop out analysis

Due to unavailability of data on the outcomes, the effective sample for the main analyses was 10,612 individuals (85% of the original sample; 82% of the women and 89% of the man). The drop out analysis found that missing women reported slightly less frequently living with children in the household (57% vs 61% *p* = 0.004), while missing men slightly more often reported to be immigrants (10% vs 7% *p* = 0.04), but with no differences with regard to any of the other sociodemographic, behavioural and psychosocial factors (all *p* values > 0.10) [26]. Altogether, there was little evidence for serious selection bias due to failure to complete the health examination, and most importantly not with respect to the key exposures.

#### Measurement of health inequalities – The concentration index (C)

To estimate socioeconomic inequalities in CVRFs (aim 1), concentration indices (C) using total earned income as the socioeconomic indicator and CVRFs as health outcomes were calculated. The concentration index (C) is a summary measure indicating whether the outcome of interest is concentrated among the population at lower or higher socioeconomic levels. The C assumes values between − 1 and + 1, if there is no inequality, it equals 0. The C is interpreted as follows: a negative concentration index (C < 0) indicates that the outcome variable is disproportionately concentrated among the poor, while a positive concentration index (C > 0) indicates the outcome variable is disproportionately concentrated among the rich.

The concentration index is formally defined as [23]:

1 $$ C=\frac{2}{n\mu}\ \sum \limits_{i=1}^n\ {h}_i{R}_i-1 $$

where *h*_*i*_ is the variable of interest for the *i*^*th*^ person; *μ* is the mean or proportion of *h*; n is the number of people; and *R*_i_ is the *i*^*th*^ ranked individual according to their socioeconomic status, from the most disadvantaged to the least disadvantaged.

An important consideration when using binary health outcomes is that the bounds of the C are not −1 and + 1 but instead depend on the mean (*μ*) of the variable [23, 39]. According to Wagstaff et al., a feasible solution to this problem is to normalize the concentration index by dividing by 1 minus the mean [23, 39]. We applied this normalization not only to the concentration index but also to the decomposition. The standard errors for the concentration indices were calculated using the delta method through the nlcom command in stata, taking into account the sampling variability of the estimated mean of the health variable.

#### Estimation of contributions of social determinates to inequalities – Decomposition analysis

To estimate the contribution of socioeconomic factors to the observed health inequalities (aim 2), Wagstaff-type decomposition analysis of concentration indices was used [23]. According to Wagstaff et al., the C can be expressed as the sum of contributions of various factors (social determinants) together with an unexplained residual component. The C can therefore be decomposed into the contributions of the *k* factors, in which each contribution is the product of the sensitivity of health with respect to *k* factors and their degree of income-related inequality [23]. Based on a linear additive regression model of health (*y*), such as:

2 $$ y=\propto +{\sum}_k{\beta}_k{x}_k+\varepsilon $$

the concentration index for *y*, *C*, can be written:

3 $$ C=\sum \limits_k\left({\beta}_k{\overline{x}}_k/\mu \right){C}_k+{GC}_{\varepsilon }/\mu $$

where *μ* is the mean of y (health outcome variable), $\overline{X}$_*k*_ is the mean of *X*_*k*_ (determinants), *C*_*k*_ is the concentration index for *X*_*k*_ (defined analogously to *C*), and *GC*_*ε*_ is the generalized concentration index for the error term (*ε*).

As the outcomes of the present study (CVRF) were non-linear, an appropriate statistical technique for non-linear settings was needed. According to the World Bank technical notes on non-linear estimation [23], one possibility is to calculate the marginal/partial effects of the *β*_*k*_ that goes in eq. 3 from a probit model and then use these marginal effects to calculate the contributions of the *k* determinants (explanatory variables) [23]. This approach of using marginal effects to calculate the non-linear estimations was therefore used in our study.

The C of each of the outcomes under analysis was decomposed by determinants in separate decomposition analyses stratified by gender. In the result sections, the contribution of each determinant to the observed health inequality is reported both as an absolute contribution (i.e. expressed in the same unit as the concentration index), and as a relative contribution (i.e. percentage of the total concentration index). The estimated marginal effects of all decomposition analyses are reported in Appendix 1.

## Results

### Characteristics of the study population

The characteristics of the study population, as well as the income inequalities (C) for each characteristic, are shown in Table 2. Women were consistently healthier and better educated than men were, but at the same time had lower income and were employed in occupations with lower status. Men engaged to a greater degree in poor health behaviours, except for smoking with similar prevalence among women and men. Furthermore, men had less availability of attachment, whereas women were more often in high strain jobs.

### Magnitude of income-related inequalities in CVRFs

**Fig. 1:**
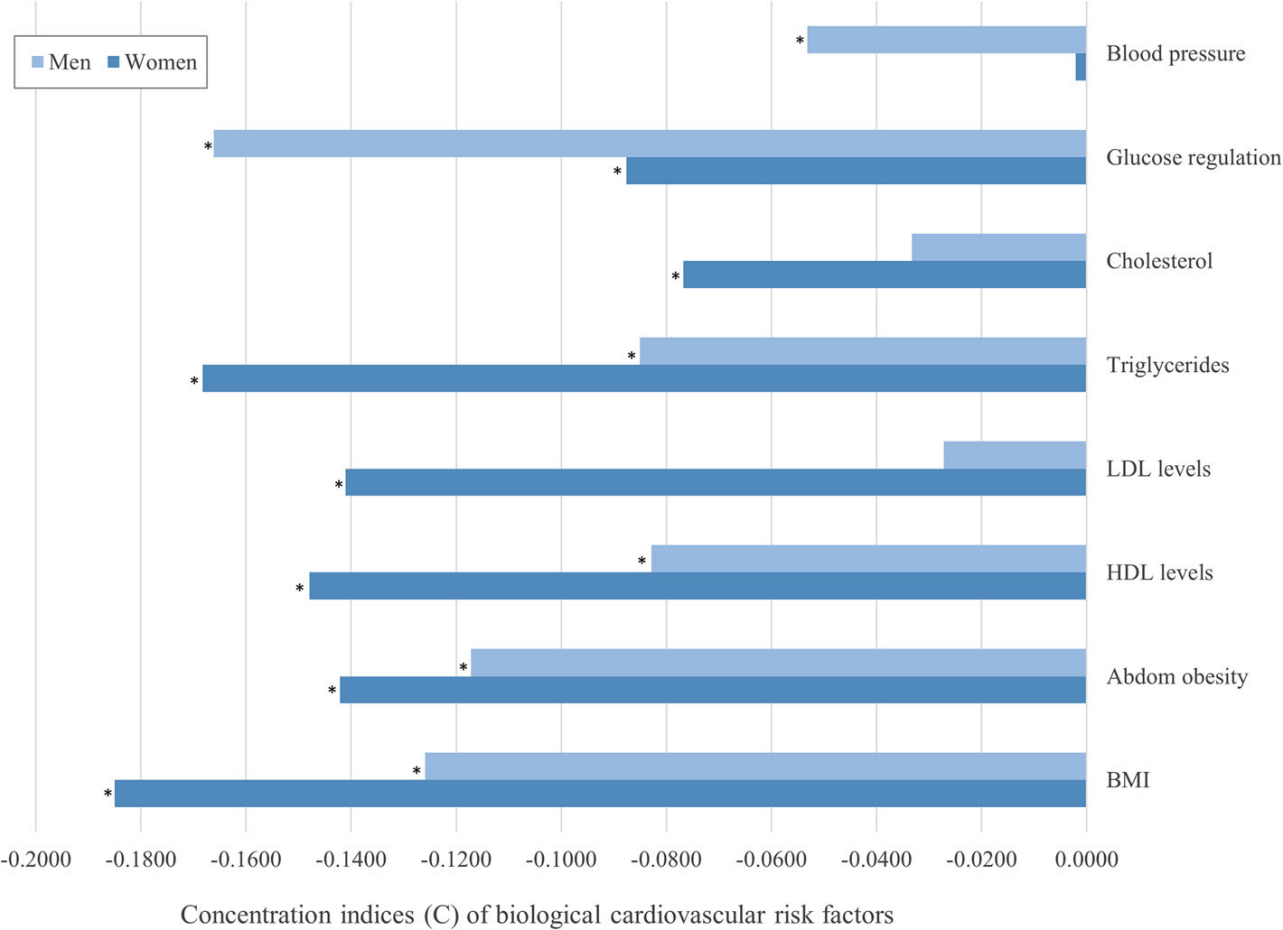
Concentration indices of cardiovascular risk factors by gender. * indicates that C differs from 0; *p* < 0.05

Directly related to aim 1, we examined the Income-related inequalities in the eight biological cardiovascular risk factors in women and men (results presented in Table 2 and Fig. 1). The concentration indices of all eight outcomes were negative for both genders, indicating that CVRFs are concentrated among the less affluent population. The magnitude of the inequalities also varied considerably, both between different CVRFs, and between genders.

Overall, income-related inequalities in CVRFs tended to be larger among women than among men, as seen for all outcomes except for glucose regulation and blood pressure. The highest inequalities among women were seen in BMI, triglycerides and HDL-cholesterol (− 0.1850 SE = 0.0192; − 0.1683 SE = 0.0215 and − 0.1479 SE = 0.0208 respectively), and all inequalities except for blood pressure were significant. Among men the largest inequalities were seen in glucose regulation, BMI and abdominal obesity (− 0.1661 SE = 0.0213; − 0.1259 SE = 0.0187 and − 0.1172 SE = 0.0164). Cholesterol and LDL inequalities in men were small and non-significant.

Concentration indices of the determinants are also presented in Table 2, as the poor-rich distributions of these factors across the income scale are key to estimate their contributions to the observed inequalities in health. In both genders, the less affluent population was concentrated among the younger age group (40 yrs), immigrants, the lower educated, low-status occupations, unmarried (women and men) and divorced (men), as well as in those residing in inland municipalities or those without a hospital. Having children in the household was concentrated among the less affluent women but among the well-off men.

Regarding behavioural factors, being physically inactive, smoking and snus use tended to be more common among less-affluent women and men, whereas harmful alcohol consumption and alcohol dependence were concentrated among the wealthier women but among the less affluent men. Of the psychosocial factors, social support (AVAT and AVSI) were more common among the better-off population while low strain, passive and high strain jobs were concentrated among the less affluent.

### Contribution of determinants to income-related inequality in CVRFs

Second, and directly corresponding to aim 2, we examined the contribution of demographic, socioeconomic, behavioural and psychosocial determinants to the observed inequalities. A summary of decomposition results for the eight CVRFs is shown in Table 3 (women) and Table 4 (men). The columns under the heading “contribution to C” and “Adj %” present absolute (in the same unit as the C) and relative (adjusted percentage contribution towards inequality) contributions of each determinant, respectively.

**Table 3:** Decomposition of income-related inequalities in cardiovascular risk factors in women aged 40- and 50-years old in 2008-2010 in Northern Sweden (*N*=5262)

	BMI		Abdominal Obesity		Cholesterol		Triglycerides		Hyperglycemia		Blood pressure		HDL levels		LDL levels	
	Cont to C	Adj %	Cont to C	Adj %	Cont to C	Adj %	Cont to C	Adj %	Cont to C	Adj %	Cont to C	Adj %	Cont to C	Adj %	Cont to C	Adj %
Demographic variables																
Age	0.0005	**0.0**	0.0133	**0.0**	0.0545	**0.0**	0.0137	**0.0**	0.0090	**0.0**	0.0537	**0.0**	-0.0187	**10.4**	0.0544	**0.0**
40 yrs																
50 yrs	0.0005		0.0133		0.0545		0.0137		0.0090		0.0537		-0.0187	10.4	0.0544	
Year of participation	0.0045	**0.3**	0.0030	**0.2**	0.0106	**1.0**	0.0012	**0.6**	0.0004	**0.3**	0.0006	**0.0**	-0.0179	**12.4**	0.0024	**0.4**
2008																
2009	-0.0007	0.3	-0.0002	0.2	-0.0012	1.0	-0.0011	0.6	-0.0003	0.3	0.0003		0.0044		-0.0006	0.4
2010	0.0051		0.0033		0.0118		0.0023		0.0007		0.0003		-0.0223	12.4	0.0030	
Subtotal	0.0050	**0.3**	0.0163	**0.2**	0.0651	**1.0**	0.0149	**0.6**	0.0094	**0.3**	0.0543	**0.0**	-0.0366	**22.8**	0.0568	**0.4**
Socioeconomic conditions																
Total earned income	-0.1007	**49.4**	-0.0544	**47.3**	-0.0373	**30.6**	-0.0791	**40.7**	-0.0189	**30.3**	0.0119	**13.0**	-0.0413	**23.0**	-0.0765	**49.1**
Lowest quintile	-0.0687	33.7	-0.0499	36.9	-0.0305	24.9	-0.0622	32.0	-0.0221	19.1	0.0185		-0.0361	20.1	-0.0499	32.1
2	-0.0276	13.5	-0.0140	10.4	-0.0019	1.5	-0.0135	7.0	-0.0128	11.1	0.0107		-0.0044	2.4	-0.0139	8.9
3	0.0000		0.0000		0.0000		0.0000	0.0	-0.0001	0.1	0.0000		0.0000		0.0001	
4	-0.0045	2.2	0.0095		-0.0050	4.1	-0.0034	1.7	0.0160		-0.0172	13.0	-0.0008	0.5	-0.0127	8.1
Highest quintile																
Education level	-0.0193	**12.1**	-0.0188	**15.8**	-0.0450	**41.0**	-0.0559	**32.0**	-0.0088	**7.8**	-0.0048	**6.4**	-0.0401	**24.3**	-0.0405	**26.3**
Compulsory education up to 9 yrs	-0.0069	3.4	-0.0058	4.3	-0.0107	8.7	-0.0197	10.1	-0.0006	0.5	-0.0058	4.4	-0.0156	8.7	-0.0099	6.4
Secondary education up to 2 yrs	-0.0147	7.2	-0.0146	10.8	-0.0351	28.7	-0.0382	19.6	-0.0080	7.0	-0.0018	1.3	-0.0231	12.9	-0.0274	17.6
Secondary education 3 yrs	-0.0031	1.5	-0.0010	0.7	-0.0043	3.5	-0.0044	2.3	0.0002		-0.0010	0.7	-0.0049	2.7	-0.0037	2.4
Post-secondary less than 3 yrs	0.0054		0.0025		0.0051		0.0063		-0.0004	0.4	0.0037		0.0035		0.0006	
Post-secondary 3 yrs or more and postgraduate																
Occupation	-0.0201	**9.8**	-0.0164	**12.2**	0.0145	**4.1**	0.0007	**3.4**	-0.0219	**19.2**	-0.0583	**47.3**	-0.0128	**7.6**	0.0025	**2.3**
Managers & Upper professionals																
Middle non-manual	-0.0015	0.7	-0.0006	0.5	-0.0051	4.1	-0.0033	1.7	0.0002		0.0041		0.0008		-0.0025	1.6
Lower non-manual	-0.0007	0.3	0.0001		0.0006		-0.0032	1.6	-0.0008	0.7	-0.0019	1.4	-0.0033	1.8	0.0016	
Skilled manual	-0.0069	3.4	-0.0105	7.8	0.0178		0.0022		-0.0085	7.4	-0.0389	29.4	-0.0057	3.2	-0.0011	0.7
Unskilled manual	-0.0110	5.4	-0.0054	4.0	0.0012		0.0050		-0.0128	11.1	-0.0217	16.4	-0.0046	2.5	0.0044	
Immigrant status	-0.0005	**0.2**	0.0014		-0.0004	0.3	-0.0012	0.6	-0.0066	5.8	0.0069		0.0001		0.0014	
Subtotal	-0.1406	**71.6**	-0.0882	**75.3**	-0.0682	**76.0**	-0.1356	**76.7**	-0.0563	**63.1**	-0.0444	**66.7**	-0.0940	**54.8**	-0.1130	**77.7**
Family conditions																
Civil status	-0.0045	**2.2**	-0.0013	**1.1**	0.0003	**0.5**	0.0006	**0.1**	-0.0027	**2.4**	-0.0032	**2.5**	-0.0008	**0.6**	-0.0005	**0.5**
Unmarried																
Married	-0.0041	2.0	-0.0011	0.8	0.0010		0.0009		-0.0023	2.0	-0.0029	2.2	-0.0005	0.3	0.0003	
Divorced	-0.0004	0.2	-0.0004	0.3	-0.0001	0.1	0.0000	0.0	-0.0003	0.3	-0.0003	0.2	0.0003		-0.0004	0.2
Widowed	0.0000	0.0	0.0002		-0.0006	0.5	-0.0002	0.1	-0.0001	0.1	0.0000	0.0	-0.0005	0.3	-0.0004	0.3
Children in household	0.0026		0.0007		0.0047		0.0042		0.0019		0.0020		0.0006		0.0046	
Subtotal	-0.0019	**2.2**	-0.0006	**1.1**	0.0050	**0.5**	0.0048	**0.1**	-0.0008	**2.4**	-0.0012	**2.5**	-0.0002	**0.6**	0.0041	**0.5**
Geographical area																
Coastal with hospital																
Coastal without hospital	-0.0061	3.0	-0.0039	2.9	-0.0028	2.3	-0.0001	0.0	0.0024		0.0008		-0.0037	2.0	-0.0046	3.0
Inland with hospital	-0.0007	0.3	-0.0001	0.1	-0.0008	0.7	-0.0004	0.2	0.0002		-0.0023	1.7	0.0001		-0.0006	0.4
Inland without hospital	-0.0051	2.5	-0.0019	1.4	-0.0088	7.2	-0.0054	2.8	0.0033		-0.0024	1.8	-0.0004	0.2	-0.0025	1.6
Other	0.0003		-0.0002	0.2	-0.0003	0.2	-0.0004	0.2	-0.0008	0.7	0.0003		-0.0002	0.1	-0.0011	0.7
Subtotal	-0.0116	**5.8**	-0.0061	**4.5**	-0.0127	**10.4**	-0.0063	**3.2**	0.0051	**0.7**	-0.0035	**3.5**	-0.0042	**2.4**	-0.0088	**5.6**
Behavioural factors																
Physical activity	-0.0333	**16.3**	-0.0199	**14.7**	-0.0108	**8.9**	-0.0165	**8.5**	-0.0209	**18.1**	-0.0146	**11.1**	-0.0177	**9.8**	-0.0056	**3.6**
Sedentary	-0.0187	9.2	-0.0099	7.3	-0.0064	5.2	-0.0097	5.0	-0.0148	12.9	-0.0088	6.7	-0.0113	6.3	-0.0021	1.4
Moderate activity	-0.0147	7.2	-0.0100	7.4	-0.0044	3.6	-0.0068	3.5	-0.0060	5.2	-0.0058	4.4	-0.0064	3.5	-0.0034	2.2
Physically active																
Smoking	-0.0004	**0.3**	-0.0005	**0.5**	-0.0025	**2.3**	-0.0101	**5.3**	-0.0026	**2.3**	0.0023	**0.0**	-0.0044	**2.7**	-0.0038	**2.7**
Never smoked																
Former smokers	0.0001		0.0003		0.0003		0.0002		0.0001		0.0000		0.0005		0.0004	
Current smokers	-0.0005	0.3	-0.0007	0.5	-0.0028	2.3	-0.0102	5.3	-0.0027	2.3	0.0023		-0.0049	2.7	-0.0041	2.7
Snus	0.0006	**0.0**	0.0001	**0.0**	-0.0004	**0.4**	0.0002	**0.0**	0.0003	**0.0**	0.0004	**0.2**	0.0004	**0.0**	-0.0003	**0.2**
Never used																
Former users	0.0004		0.0001		-0.0004	0.3	0.0002		0.0001		-0.0003	0.2	0.0000		-0.0003	0.2
Current users	0.0002		0.0000		-0.0001	0.0	-0.0001	0.0	0.0001		0.0007		0.0003		0.0000	0.0
Alcohol disorder test	0.0000	**0.0**	0.0002	**0.0**	0.0000	**0.0**	0.0010	**0.0**	0.0004	**0.0**	0.0009	**0.0**	-0.0008	**0.5**	-0.0006	**0.4**
Not at risk																
Probably risk alcohol consumption	-0.0001	0.0	0.0002		0.0000	0.0	0.0009		0.0002		0.0009		-0.0008	0.5	-0.0006	0.4
Probably alcohol dependence	0.0000		0.0000		0.0000		0.0001		0.0002		0.0000	0.0	0.0000	0.0	0.0000	0.0
Subtotal	-0.0331	**16.6**	-0.0201	**15.3**	-0.0138	**11.5**	-0.0254	**13.8**	-0.0228	**20.5**	-0.0110	**11.3**	-0.0225	**13.0**	-0.0103	**6.9**
Psychosocial factors																
Availability of social interaction	0.0094		0.0017		-0.0008	**0.6**	-0.0057	**2.9**	-0.0105	**9.1**	-0.0065	**4.9**	-0.0015	**0.8**	-0.0040	**2.6**
Availability of attachment	-0.0016	**0.8**	-0.0018	**1.3**	0.0008		0.0059		0.0112		-0.0035	**2.7**	0.0019		0.0007	
Job strain	-0.0027	**2.7**	-0.0014	**2.3**	0.0142	**0.0**	0.0052	**2.6**	-0.0030	**3.9**	-0.0017	**8.4**	0.0049	**5.5**	0.0075	**6.3**
Low strain																
Passive	0.0025		0.0011		0.0063		-0.0049	2.5	-0.0037	3.2	0.0061		0.0101		0.0159	
Active	-0.0054	2.7	-0.0031	2.3	0.0074		0.0102		0.0016		-0.0111	8.4	-0.0100	5.5	-0.0098	6.3
High strain	0.0002		0.0006		0.0005		-0.0001	0.1	-0.0008	0.7	0.0034		0.0047		0.0014	
Subtotal	0.0052	**3.4**	-0.0015	**3.6**	0.0142	**0.6**	0.0053	**5.5**	-0.0023	**13.1**	-0.0116	**16.0**	0.0053	**6.4**	0.0041	**8.9**
Inequality (total)	**-0.1850**		**-0.1421**		**-0.0767**		**-0.1683**		**-0.0876**		-0.0021		**-0.1479**		**-0.1410**	
Standard error	0.0192		0.0148		0.0266		0.0215		0.0205		0.0237		0.0208		0.0352	
Residual	-0.0080		-0.0419		-0.0664		-0.0261		-0.0199		0.0153		0.0043		-0.0739	

*BMI* Body Mass Index, *LDL* Low-Density Lipoprotein Cholesterol, *HDL* High-Density Lipoprotein Cholesterol, *Cont to C* Contribution to C, *Adj %* Adjusted percentage. Bold numbers indicate relative contribution per variable; relative contribution per group of variables (Subtotal); and significant (p<0.05) concentration indices (Inequality (total)

**Table 4:** Decomposition of income-related inequalities in cardiovascular risk factors in men aged 40- and 50-years old in 2008–2010 in Northern Sweden (*N* = 5350)

	BMI		Abdominal Obesity		Cholesterol		Triglycerides		Hyperglycemia		Blood pressure		HDL levels		LDL levels	
	Cont to C	Adj %	Cont to C	Adj %	Cont to C	Adj %	Cont to C	Adj %	Cont to C	Adj %	Cont to C	Adj %	Cont to C	Adj %	Cont to C	Adj %
Demographic variables																
Age	-0.0011	**0.7**	0.0019	**0.0**	0.0068	**0.0**	0.0006	**0.0**	0.0080	**0.0**	0.0119	**0.0**	-0.0034	**2.7**	0.0070	**0.0**
40 yrs																
50 yrs	-0.0011	0.7	0.0019		0.0068		0.0006		0.0080		0.0119		-0.0034	2.7	0.0070	
Year of participation	0.0018	**0.5**	0.0033	**0.4**	0.0089	**0.8**	0.0005	**1.7**	0.0011	**0.0**	0.0054	**0.1**	-0.0192	**20.1**	0.0070	**0.5**
2008																
2009	-0.0008	0.5	-0.0006	0.4	-0.0008	0.8	-0.0016	1.7	0.0004		-0.0001	0.1	0.0067		-0.0005	0.5
2010	0.0026		0.0039		0.0097		0.0021		0.0007		0.0055		-0.0259	20.1	0.0075	
Subtotal	0.0007	**1.2**	0.0052	**0.4**	0.0157	**0.8**	0.0011	**1.7**	0.0090	**0.0**	0.0173	**0.1**	-0.0226	**22.8**	0.0141	**0.5**
Socioeconomic conditions																
Total earned income	-0.0430	**27.4**	-0.0354	**26.8**	-0.0101	**12.4**	-0.0162	**23.2**	-0.0998	**50.0**	-0.0368	**26.2**	-0.0140	**14.5**	-0.0062	**13.1**
Lowest quintile	-0.0330	21.0	-0.0282	21.2	-0.0035	3.4	-0.0214	23.2	-0.0809	37.5	-0.0275	17.5	-0.0077	6.0	0.0029	
2	-0.0031	2.0	-0.0034	2.5	0.0026		0.0024		-0.0268	12.4	-0.0136	8.7	0.0047		0.0041	
3	0.0000	0.0	0.0000		0.0000	0.0	0.0000		-0.0002	0.1	0.0000	0.0	0.0000		0.0002	
4	-0.0069	4.4	-0.0039	3.0	-0.0092	9.0	0.0027		0.0082		0.0044		-0.0110	8.5	-0.0135	13.1
Highest quintile																
Education level	-0.0310	**25.6**	-0.0256	**24.6**	-0.0161	**21.2**	-0.0178	**22.2**	-0.0187	**13.7**	-0.0310	**23.9**	-0.0205	**15.9**	-0.0116	**16.0**
Compulsory education up to 9 yrs	-0.0100	6.4	-0.0065	4.9	-0.0015	1.5	-0.0059	6.3	-0.0083	3.8	-0.0096	6.1	-0.0050	3.9	-0.0050	4.9
Secondary education up to 2 yrs	-0.0281	17.9	-0.0246	18.5	-0.0202	19.8	-0.0137	14.9	-0.0201	9.3	-0.0266	16.9	-0.0138	10.7	-0.0115	11.1
Secondary education 3 yrs	-0.0020	1.3	-0.0015	1.1	0.0000	0.0	-0.0010	1.0	-0.0011	0.5	-0.0013	0.8	-0.0014	1.1	0.0000	0.0
Post-secondary less than 3 yrs	0.0091		0.0070		0.0056		0.0027		0.0108		0.0065		-0.0004	0.3	0.0049	
Post-secondary 3 yrs or more and postgraduate																
Occupation	-0.0069	**4.5**	-0.0052	**4.4**	0.0181	**0.4**	-0.0032	**3.5**	-0.0147	**6.9**	-0.0116	**13.2**	0.0208	**4.2**	-0.0016	**12.2**
Managers & Upper professionals																
Middle non-manual	-0.0040	2.5	-0.0045	3.4	0.0062		0.0000		-0.0009	0.4	0.0091		-0.0054	4.2	0.0110	
Lower non-manual	-0.0006	0.4	-0.0001	0.0	-0.0004	0.4	-0.0009	1.0	0.0002		-0.0024	1.5	0.0019		-0.0002	0.2
Skilled manual	-0.0024	1.5	0.0006		0.0115		-0.0007	0.8	-0.0041	1.9	-0.0175	11.1	0.0209		-0.0105	10.2
Unskilled manual	0.0000		-0.0012	0.9	0.0008		-0.0015	1.7	-0.0099	4.6	-0.0009	0.6	0.0033		-0.0018	1.8
Immigrant status	0.0013		0.0006		0.0015		-0.0018	**1.9**	-0.0043	**2.0**	0.0027		-0.0049	**3.8**	0.0025	
Subtotal	-0.0796	**57.5**	-0.0656	**55.7**	-0.0065	**34.0**	-0.0390	**50.8**	-0.1374	**72.6**	-0.0767	**63.3**	-0.0187	**38.5**	-0.0169	**41.3**
Family conditions																
Civil status	-0.0069	**5.0**	-0.0098	**8.0**	-0.0088	**10.4**	-0.0020	**2.3**	-0.0144	**7.2**	-0.0023	**2.1**	-0.0052	**4.3**	-0.0025	**4.6**
Unmarried																
Married	-0.0078	5.0	-0.0106	8.0	-0.0106	10.4	-0.0020	2.2	-0.0156	7.2	-0.0032	2.0	-0.0056	4.3	-0.0048	4.6
Divorced	0.0005		0.0004		0.0012		-0.0001	0.1	0.0012		0.0010		0.0002		0.0017	
Widowed	0.0004		0.0004		0.0006		0.0001		0.0000	0.0	-0.0001	0.1	0.0001		0.0005	
Children in household	-0.0098	**6.2**	-0.0086	**6.5**	-0.0140	**13.7**	-0.0094	**10.2**	-0.0090	**4.2**	-0.0165	**10.5**	-0.0026	**2.0**	-0.0128	**12.4**
Subtotal	-0.0167	**11.2**	-0.0184	**14.5**	-0.0227	**24.1**	-0.0114	**12.4**	-0.0234	**11.4**	-0.0188	**12.6**	-0.0078	**6.3**	-0.0153	**17.0**
Geographical area																
Coastal with hospital																
Coastal without hospital	-0.0029	1.9	-0.0040	3.0	-0.0068	6.7	-0.0004	0.5	0.0031		-0.0063	4.0	-0.0023	1.8	-0.0045	4.4
Inland with hospital	-0.0009	0.6	-0.0003	0.3	-0.0016	1.5	0.0002		-0.0004	0.2	-0.0023	1.5	-0.0001	0.1	-0.0011	1.1
Inland without hospital	-0.0047	3.0	-0.0010	0.7	-0.0087	8.5	-0.0016	1.8	0.0050		-0.0112	7.1	-0.0056	4.4	-0.0107	10.4
Other	-0.0001	0.1	-0.0002	0.1	0.0001		-0.0003	0.3	0.0000	0.0	0.0000		-0.0014	1.1	0.0001	
Subtotal	-0.0086	**5.5**	-0.0055	**4.2**	-0.0170	**16.8**	-0.0022	**2.6**	0.0076	**0.2**	-0.0198	**12.6**	-0.0094	**7.3**	-0.0162	**15.8**
Behavioural factors																
Physical activity	-0.0224	**14.3**	-0.0192	**14.5**	-0.0079	**7.7**	-0.0150	**16.3**	-0.0140	**6.5**	-0.0140	**8.9**	-0.0250	**19.4**	-0.0098	**9.5**
Sedentary	-0.0166	10.6	-0.0136	10.3	-0.0051	5.0	-0.0105	11.4	-0.0096	4.5	-0.0129	8.2	-0.0147	11.4	-0.0053	5.1
Moderate activity	-0.0058	3.7	-0.0056	4.2	-0.0028	2.8	-0.0045	4.9	-0.0043	2.0	-0.0011	0.7	-0.0103	8.0	-0.0045	4.4
Physically active																
Smoking	-0.0068	**4.4**	-0.0058	**4.3**	-0.0051	**5.0**	-0.0074	**8.0**	-0.0044	**2.0**	0.0043	**0.0**	-0.0049	**3.8**	0.0000	**0.2**
Never smoked																
Former smokers	-0.0032	2.0	-0.0016	1.2	-0.0012	1.2	-0.0008	**0.8**	-0.0022	1.0	0.0015		-0.0004	**0.3**	-0.0002	**0.2**
Current smokers	-0.0037	2.4	-0.0041	3.1	-0.0039	3.8	-0.0066	7.2	-0.0022	1.0	0.0028		-0.0046	3.6	0.0002	
Snus	-0.0007	**0.5**	-0.0005	**0.8**	-0.0006	**2.1**	-0.0015	**1.8**	0.0003	**0.0**	0.0003	**0.0**	-0.0001	**0.5**	-0.0003	**1.2**
Never used																
Former users	0.0001		0.0006		0.0015		0.0002		0.0000		0.0001		-0.0007	0.5	0.0009	
Current users	-0.0008	0.5	-0.0010	0.8	-0.0021	2.1	-0.0016	1.8	0.0002		0.0002		0.0006		-0.0013	1.2
Alcohol disorder test	-0.0008	**0.5**	-0.0009	**0.7**	-0.0021	**2.0**	-0.0005	**0.8**	-0.0015	**0.7**	-0.0015	**0.9**	0.0029	**0.0**	-0.0027	**2.6**
Not at risk																
Probably risk alcohol consumption	-0.0007	**0.5**	-0.0009	**0.7**	-0.0009	0.9	-0.0008	0.8	-0.0001	0.0	-0.0008	0.5	0.0014		-0.0007	0.7
Probably alcohol dependence	0.0000	0.0	0.0000		-0.0012	1.1	0.0002		-0.0014	0.7	-0.0006	0.4	0.0014		-0.0020	2.0
Subtotal	-0.0308	**19.7**	-0.0263	**20.3**	-0.0157	**16.9**	-0.0245	**26.9**	-0.0196	**9.2**	-0.0108	**9.8**	-0.0272	**23.8**	-0.0129	**13.6**
Psychosocial factors																
Availability of social interaction	0.0106		-0.0031	**2.3**	-0.0004	**0.4**	0.0078		0.0046		0.0093		0.0042		-0.0042	**4.0**
Availability of attachment	-0.0018	**1.2**	-0.0010	**0.7**	-0.0006	**0.6**	-0.0010	**1.1**	-0.0008	**0.4**	0.0022		-0.0006	**0.5**	-0.0003	**0.3**
Job strain	0.0048	**3.8**	0.0047	**1.8**	0.0063	**6.6**	0.0037	**4.5**	0.0011	**6.2**	0.0111	**1.6**	-0.0007	**0.7**	0.0149	**7.6**
Low strain																
Passive	-0.0020	1.3	0.0011		-0.0047	4.6	-0.0019	2.1	-0.0090	4.2	0.0027		-0.0009	0.7	-0.0039	3.8
Active	0.0107		0.0060		0.0130		0.0079		0.0146		0.0110		0.0000	0.0	0.0227	
High strain	-0.0039	2.5	-0.0024	1.8	-0.0020	2.0	-0.0023	2.5	-0.0045	2.1	-0.0025	1.6	0.0002		-0.0039	3.7
Subtotal	0.0135	**5.0**	0.0006	**4.9**	0.0053	**7.5**	0.0105	**5.6**	0.0050	**6.6**	0.0227	**1.6**	0.0028	**1.2**	0.0104	**11.9**
Inequality (total)	**-0.1259**		**-0.1172**		-0.0333		**-0.0850**		**-0.1661**		**-0.0531**		**-0.0828**		-0.0272	
Standard error	0.0187		0.0164		0.0214		0.0159		0.0213		0.0194		0.0230		0.0253	
Residual	-0.0043		-0.0071		0.0077		-0.0195		-0.0074		0.0330		0.0004		0.0096	

*BMI* Body Mass Index, *LDL* Low-Density Lipoprotein Cholesterol, *HDL* High-Density Lipoprotein Cholesterol, *Cont to C* Contribution to C, *Adj %* Adjusted percentage. Bold numbers indicate relative contribution per variable; relative contribution per group of variables (Subtotal); and significant (p<0.05) concentration indices (Inequality (total)

Overall, socioeconomic conditions and health behaviours were the factors that played the largest role in explaining income inequalities across the eight CVRFs in both women and men. However, the contributions of specific factors differed between the CVRFs and with both similarities and differences between genders.

In women, socioeconomic conditions were the main contributors explaining from 54.8 to 76.7% of the inequalities (Table 3). Income was the dominant factor for BMI, abdominal obesity, triglycerides, glucose regulation and LDL-cholesterol, explaining between 30 and 49% of the inequality, whereas education was more important for HDL-cholesterol and total-cholesterol (explaining 24.3 and 41.0% respectively), and occupation was more important for blood pressure (explaining 47.3%). Behavioural factors came next in independent explanatory importance with contributions between 6.9 to 20.5% depending on CVRF, with physical activity being the most important contributor among this group of factors (3.6 to 16.3% contribution). Smoking, snus use and alcohol consumption made insubstantial independent contributions to explain the observed inequalities.

Psychosocial factors were the third most important set of factors, jointly contributing to a moderate degree to the inequalities in triglycerides, glucose regulation, blood pressure, HDL-cholesterol and LDL-cholesterol (explaining 5.5–16.0%), whereas for BMI, abdominal obesity and total-cholesterol, geographical area was the third most important explaining factor. Demographics and family conditions made small independent contributions (less than 2.5%) to all of the observed health inequalities in women, except for HDL-cholesterol where the year of participation and age together contributed 22.8% of the explanation.

The corresponding analyses in men (Table 4) showed similarly that socioeconomic conditions contributed strongly to health inequalities (34.0–72.6% contribution), although overall, the magnitude of contribution was smaller than in women. Similar to women, income was the dominant factor for BMI, abdominal obesity, triglycerides, glucose regulation and blood pressure explaining between 23.2 to 50.0% of the inequality, whereas education was more important for total-cholesterol, HDL and LDL.

The explanatory role of behavioural factors was greater (9.2 to 26.9%) in men than it was for women. Similar to women, physical activity was the most important behavioural factor explaining 7.7 to 19.4% of the inequality. In contrast to women, smoking, snus use and alcohol consumption played a greater, but still moderate, explanatory role. Interestingly, family conditions (civil status and having children in the household) were more important in men than in women, this group of variables was third in importance and contributed to a moderate degree to inequalities in all CVRFs (from 6 to 24%).

Geographical area came next in explanatory role, contributing to a moderate degree to inequalities in BMI, total-cholesterol, blood pressure, HDL-cholesterol and LDL-cholesterol (explaining 5.5–16.8%), whereas for abdominal obesity, triglycerides and glucose regulation, the psychosocial factors were next in level of importance. Similar to women, the demographic factors made insubstantial contributions (less than 2% contribution) to all of the observed health inequalities except for HDL-cholesterol.

As an overall assessment of explanatory strengths of the decomposition models, most of the inequality in CVRFs to the disadvantage of the less affluent segment of the population was explained by the determinants observed in this study, as seen in the small residuals. Exceptions were blood pressure in women, total-cholesterol and LDL-cholesterol in men in which the inequalities were small and the concentration index non-significant. The decomposition estimates from all eight CVRFs are reported as a point of reference, but the estimates from the mentioned non-significant concentration indices should be interpreted carefully.

## Discussion

The present study of a middle-aged Northern Swedish population demonstrated firstly, substantial income-related inequalities in CVRFs which differ in magnitude. Moreover, despite better cardiovascular health in women during middle age, women displayed greater inequalities than did men with respect to most risk factors, except diabetes and blood pressure. Second, for both women and men, socioeconomic conditions and health behaviours were the most important factors explaining inequalities in all CVRFs. However, patterns of dominant explanatory factors differed between genders; whereas tobacco use and alcohol consumption made insubstantial contributions in women, they played a greater although moderate role in men. Family conditions were more important for men, whereas psychosocial factors were more important for women’s health inequality.

Socioeconomic inequalities in CVRFs, including obesity, hypertension, diabetes and raised cholesterol, have generally been found among the poor [5, 7, 18–21], although some studies report obesity and alcohol consumption to concentrate among well-off populations [17, 24, 25]. Our findings overall confirm substantial income-related inequalities in multiple CVRFs to the disadvantage of the less affluent in Sweden, which expands previous Swedish research focusing on specific factors such as obesity [21] or educational inequalities in various CVRFs [11, 13, 14]. Previous studies have pointed out that increased income-related inequalities in cardiovascular disease or behavioural risk factors coincide with increased income inequalities in Sweden [3, 16, 40], which together could be seen as possible consequences of the declining welfare state [41].

While there is a considerable body of research implemented in guidelines for CVD prevention aiming to identify which (or what combination of) CVRFs are most predictive of CVD and as such should be targeted for intervention [5], there are no similar developments when it comes to preventing an unequal social distribution of CVD. To this end, very unequally distributed CVRFs have the potential to be more important precursors to inequalities in CVD, while CVRFs with small inequalities cannot realistically contribute substantially to CVD inequalities, even if they are strong risk factors for CVD itself. From our study, the magnitude of inequalities in the different CVRFs implies that certain risk factors, e.g. obesity both for men and women, blood lipids in women and glucose regulation in men, may play a more important role in the rising socioeconomic inequalities in cardiovascular morbidity and mortality. As a corollary, preventive efforts may have greater prospects of specifically reducing inequalities in CVD if targeting these factors rather than factors with smaller inequalities, e.g. blood pressure in women and cholesterol/LDL levels in men.

Similar to our findings most of the studies from developed countries have shown that, despite men having a worse cardiovascular profile, women present larger inequalities in the CVRFs [5, 7, 10, 19–21]. This picture illustrates the complex role of gender when it comes to income-related inequalities, and mirrors the worrying developments in health and life expectancy specifically for socioeconomically disadvantaged women in Sweden [2]. For the particular case of VIP, women were shown to be healthier and displayed greater reductions in risk factors and increasing awareness of control and treatment compared to men [11–13, 15], but at the same time they have occupations with lower status and lesser earnings than their male counterparts, which creates a relative disadvantage. Furthermore, the overall higher inequalities in women emphasize the need for structural policies to equalize income between genders. As such, the present study highlights the need for health preventive efforts with a gender and equity lens to focus on socioeconomically vulnerable women.

Our study also shows that the observed health inequalities can be explained by factors of general importance, but also that the patterns of importance may differ between genders and specific CVRFs. First, socioeconomic conditions were important factors explaining the inequalities in both genders and for all CVRFs under analysis. Income inequality was the factor that explains the largest fraction of the inequalities, but with a greater explanatory role in women than in men. Education level was the second largest contributor but contrary to income, this factor played a greater role in men than in women. Previous decomposition analyses on single cardiovascular risk factors (obesity, alcohol consumption) conducted in Sweden [21, 24], and in other contexts [17–20] have also found that socioeconomic inequalities explain the largest fraction of health inequalities. The differential roles of income and education between women and men have also been identified before [19, 20, 24], and illustrate once again the relative disadvantage of women in the labour market expressed by their lower incomes. The dominant focus of educational rather than income inequalities in Swedish research as well as governmental reports [2] might thus misrepresent structural inequalities for women which is only partially attributable to educational inequalities. Overall, these findings suggest that health inequalities will be difficult to address without addressing the roots of the problem – entangled income and gender inequalities in Swedish society.

Second, the magnitude of the contribution of behavioural factors was greater in men than in women, with physical activity being the most important contributor for both genders and tobacco use and alcohol consumption displaying small contributions in men but not in women. The overall small contribution of smoking to the inequalities is expected and should not be interpreted as smoking not playing a role in inequalities in manifest CVD morbidity or mortality, since smoking acts on CVD through chiefly other biological mechanisms than those captured in this study, e.g. by inducing atherosclerosis development and thrombotic phenomena [42]. Somewhat contrasting patterns to those found in the present study for smoking and alcohol consumption have however been described in other contexts, e.g. being more important in explaining inequalities in obesity among women than in men [17], or contributing to health inequalities in disparate directions [19]. Recognizing this diversity in patterns, our findings suggest that in this Northern Swedish context, addressing inequalities in physical activity may be a moderately effective strategy to reduce health inequalities, especially since physical activity has a positive effect on a greater range of metabolic CVRFs than those measured in this study [42]. One example is the VIP programme, which on a population basis and integrated into routine primary care targets e.g. obesity and physical inactivity, and which has shown reductions in both all-cause and CVD mortality in all educational groups [43].

Third, psychosocial factors and family conditions were of moderate importance to inequalities in CVRFs, with psychosocial factors of greater importance for women and family conditions more important for men. Other cross-sectional decomposition analyses have similarly estimated small to moderate contributions of family factors [17, 19, 21, 25], but also that single marital status can be of greater importance for women’s obesity inequalities and in either a supporting [17] or counteracting [21] role. Psychosocial contributions to inequalities in CVRFs have to our knowledge not been examined previously in decomposition analysis, although there is some evidence of people with high job strain being more likely to have diabetes, to smoke, be physically inactive and obese [44, 45], but with a debated impact on CVD and no association demonstrated in the VIP population [37]. Social support was shown to be a protective factor for some CVRFs, particularly health behaviours [46, 47], but as in job strain, evidence regarding differential contributions by gender is scarce. In summary, inequalities in psychosocial and family conditions seem to play a moderately important role, but with gendered patterns. The different gender patterns of contribution identified in our study suggest different mechanisms through which family and psychosocial factors can shape inequalities. The complexity of theses influences merit further analysis.

Fourth, geographical location and demographic factors were of low to moderate importance in explaining inequalities, irrespective of gender. Other studies have also shown similar low-moderate contributions when analysing urban/rural areas [17, 19]. Although other studies generally have found that age contributes to health inequalities, such patterns would be difficult to find in the present sample that was very homogenous with respect to age. Moreover, as the estimates are adjusted for all other factors, this does not necessarily mean that geographical and demographic factors are unimportant [11], but alternatively that their contribution is attributed to other factors included in the model, e.g. geographical location might be explained by the income differences between the areas.

Lastly, residuals for the decomposition models were small indicating that most of the inequalities in both women and men were well explained by the observed factors. However, the slightly larger residuals in women suggest that other social determinants not included in this analysis may contribute to women’s health inequalities. The complexity and influence of other factors such as the unequal distribution of domestic work and other conditions related to gender equality should be added in future analysis to better capture the underpinnings of health inequalities in women. For example, as some studies suggest a greater influence of early life course socioeconomic conditions on adult women’s obesity [48], early life course conditions could be one possible source of health inequalities in women.

### Methodological considerations

The main strengths of the present study are the large sample, the use of a comprehensive set of outcome measures and explanatory factors using a combination of register, survey and measured data, and the use of a novel statistical method.

Some potential limitations should be considered when interpreting our results. The population in our study comes from participants in VIP, and as such, it is a sample of the total population of Västerbotten aged 40 or 50 yrs. in 2008–2010. Examinations of participation in the VIP have found a decreasing but present, slight underrepresentation of men, immigrants, singles and poorer people compared to the target population [28], which suggests a possible measure of selection bias. However, the median income of the present sample only differed by < 2% from official statistics of the population of Västerbotten for both women and men, indicating that this central indicator was fairly representative of the target population. While selection bias is highly problematic, e.g. for prevalence estimates, it may be less likely that this leads to seriously biased estimates of associations or concentrations as was the focus of the present study.

Overall, the measures are deemed good; the outcome measures were all taken during health examinations following standard procedures [27]; the health behaviours and psychosocial factors were measured by established or validated instruments [27]; and the socioeconomic and demographic factors were retrieved from the high-quality Swedish total population registers. However, the income variable only comprises individually earned income and as such does not reflect other aspects of the total financial situation such as wealth. It is likely that using a different or more comprehensive measure of income would have display a different ranking and consequently a different level of inequalities than those reported in this study. Although the range of determinants was limited by those routinely collected in national registers and the VIP questionnaire, we included a more comprehensive set of determinants than previous decomposition has done. Nevertheless, unmeasured factors could change the inferences. For example, even though diet has been recognized as a health behaviour related to inequalities in most CVRFs [42, 49], the food frequency questionnaire in VIP was not available. Relatedly, since the present study focused on biological CVRFs, health behaviours like smoking were therefore designated as behavioural determinants, however they can also be seen as independent CVRFs in themselves.

Concerning the analysis, decomposition of the concentration indices can be viewed as a useful method to identify factors lying behind health inequality. However, it cannot provide causal inference and does not identify mediating pathways [23], a matter that is only made more challenging by the cross-sectional nature of our data. Moreover, decomposition analysis can only handle a single outcome and as such cannot take interrelationships between the CVRF outcomes into account. Another recognized limitation of decomposition analysis is that the method relies on linear models; in our case, all outcomes were binary, therefore we applied Wagstaff correction [23, 39] for both calculation of the concentration index and decomposition analysis. It should be noted that there are other correction alternatives when dealing with binary outcomes [50], which could possibly yield different inferences.

## Conclusion

Taken together, the present study demonstrates clear income inequalities in a broad range of CVRFs in a Swedish middle-aged population, with overall greater inequalities in women. Findings suggest that the magnitude of income-related inequalities in CVRFs and their determinants differ importantly between the risk factors and gender, a variation that should be taken into consideration in population interventions aiming to prevent inequalities in manifest CVD. Focusing on the larger inequalities in CVRFs, such as obesity, and by targeting modifiable factors of broad importance, such as inequalities in physical activity, may contribute to a reduction in inequalities in cardiovascular health. Nevertheless, the results also suggest that without addressing the root causes of socioeconomic inequalities, including income inequalities and the structural disadvantage of women, health inequalities will endure, particularly in women.

## Appendix 1

**Table 5:** Marginal effects from the probit models of the eight biological cardiovascular risk factors in VIP participants aged 40- and 50-years old in 2008–2010 by gender

Variable dF/dx	BMI		Abdominal Obesity		Cholesterol		Triglycerides		Hyperglycemia		Blood pressure		HDL levels		LDL levels	
	Women	Men	Women	Men	Women	Men	Women	Men	Women	Men	Women	Men	Women	Men	Women	Men
Age																
40 years																
50 years	0.002	-0.016	**0.094**	**0.046**	**0.079**	**0.073**	**0.034**	0.015	**0.024**	**0.090**	**0.100**	**0.162**	**-0.090**	**-0.047**	**0.045**	**0.058**
Year of participation																
2008																
2009	**0.028**	**0.023**	0.024	0.023	**0.021**	0.015	**0.032**	**0.067**	0.012	-0.008	-0.007	0.003	**-0.074**	**-0.089**	0.006	0.006
2010	**0.038**	**0.025**	**0.055**	**0.054**	**0.040**	**0.063**	0.013	**0.033**	0.005	0.006	0.001	**0.046**	**-0.131**	**-0.129**	0.006	**0.033**
Total earned income																
Lowest quintile	**0.061**	0.034	**0.099**	**0.042**	0.012	0.002	**0.042**	0.036	0.016	**0.063**	-0.010	0.025	0.044	0.007	0.011	-0.001
2	**0.050**	0.006	**0.057**	0.010	0.002	-0.004	0.019	-0.008	0.019	**0.041**	-0.011	0.025	0.011	-0.009	0.006	-0.004
3	0.018	-0.009	**0.038**	-0.019	-0.010	-0.005	0.015	-0.015	0.009	0.023	-0.012	-0.014	0.011	0.004	-0.005	-0.011
4	-0.008	-0.014	**0.038**	-0.012	-0.004	-0.013	-0.004	0.009	0.025	0.013	-0.018	0.008	-0.003	-0.021	-0.006	-0.013
Highest quintile																
Education level																
Compulsory education up to 9 yrs	**0.053**	**0.089**	**0.097**	**0.086**	**0.037**	0.009	**0.116**	**0.085**	0.004	**0.055**	0.025	**0.076**	**0.152**	0.039	0.020	0.021
Secondary education up to 2 yrs	**0.030**	**0.076**	**0.067**	**0.099**	**0.032**	**0.038**	**0.060**	**0.061**	0.014	**0.040**	0.002	**0.064**	**0.065**	0.036	**0.015**	0.015
Secondary education 3 yrs	**0.041**	**0.079**	0.027	**0.087**	**0.025**	0.000	**0.043**	**0.062**	-0.003	0.035	0.007	**0.045**	**0.103**	**0.060**	0.013	0.000
Post-secondary less than 3 yrs	**0.048**	**0.068**	**0.049**	**0.080**	**0.020**	0.029	**0.043**	0.034	-0.003	**0.062**	0.019	**0.044**	**0.040**	-0.003	0.001	0.018
Post-secondary 3 yrs or more and postgraduate																
Occupation																
Managers & Upper professionals																
Middle non-manual	-0.009	-0.018	-0.008	-0.031	-0.013	0.020	-0.015	0.000	0.001	-0.003	0.014	**0.037**	0.007	-0.022	-0.004	**0.025**
Lower non-manual	0.007	0.016	-0.002	0.002	-0.003	0.007	0.025	0.039	0.007	-0.003	0.011	**0.053**	0.046	-0.055	-0.004	0.003
Skilled manual	0.010	0.003	0.034	-0.001	-0.012	-0.010	-0.002	0.001	0.011	0.004	**0.033**	0.019	0.012	-0.024	0.000	0.006
Unskilled manual	**0.061**	-0.001	**0.066**	0.019	-0.003	-0.005	-0.021	0.026	**0.059**	**0.078**	**0.068**	0.008	0.035	-0.032	-0.006	0.009
Immigrant status	0.004	-0.014	-0.027	-0.010	0.002	-0.011	0.009	0.032	**0.051**	**0.037**	**-0.034**	-0.026	-0.002	**0.053**	-0.003	-0.014
Civil status																
Unmarried																
Married	**-0.027**	-0.016	-0.016	**-0.033**	0.003	-0.015	0.004	-0.007	-0.012	**-0.024**	-0.011	-0.006	-0.004	-0.011	0.001	-0.005
Divorced	**-0.038**	-0.014	**-0.071**	-0.016	-0.003	-0.023	-0.001	0.003	-0.024	-0.025	-0.014	-0.023	-0.017	-0.005	-0.010	-0.021
Widowed	-0.004	0.093	0.040	0.162	-0.027	0.113	-0.015	0.030	-0.010	-0.004	-0.001	-0.022	-0.056	0.040	-0.012	0.063
Children in household	**-0.025**	-**0.027**	-0.017	**-0.034**	**-0.020**	**-0.026**	**-0.032**	**-0.042**	-0.018	**-0.018**	-0.012	**-0.039**	-0.008	-0.006	**-0.012**	**-0.017**
Geographical area																
Coastal with hospital																
Coastal without hospital	**0.063**	**0.030**	**0.088**	**0.061**	0.012	**0.049**	0.001	0.007	-0.021	**-0.025**	-0.005	**0.057**	**0.041**	0.019	**0.012**	**0.023**
Inland with hospital	**0.055**	**0.073**	0.017	0.038	**0.036**	**0.085**	**0.036**	-0.020	-0.013	0.025	**0.104**	**0.164**	-0.017	0.007	0.015	**0.049**
Inland without hospital	**0.050**	**0.041**	**0.042**	0.013	**0.039**	**0.053**	**0.041**	0.024	**-0.028**	**-0.033**	0.014	**0.088**	0.006	**0.045**	0.007	**0.044**
Other	-0.057	0.035	0.101	0.078	0.030	-0.015	0.072	0.120	**0.149**	-0.007	-0.043	-0.004	0.103	**0.319**	0.063	-0.016
Physical activity																
Sedentary	**0.148**	**0.098**	**0.172**	**0.119**	**0.022**	0.021	**0.056**	**0.103**	**0.098**	**0.044**	**0.040**	**0.068**	**0.119**	**0.086**	0.004	0.015
Moderate activity	**0.080**	**0.064**	**0.121**	**0.093**	0.011	**0.022**	**0.029**	**0.081**	**0.025**	**0.038**	**0.018**	0.012	**0.050**	**0.075**	0.005	**0.024**
Physically active																
Smoking																
Never smokers																
Former smokers	0.014	**0.040**	**0.043**	**0.032**	**0.020**	0.011	0.015	0.016	0.007	**0.021**	0.002	-0.018	0.022	0.003	**0.012**	0.001
Smokers	0.006	**0.030**	0.019	**0.051**	0.015	0.023	**0.091**	**0.092**	**0.026**	0.014	-0.015	-0.020	**0.068**	**0.039**	**0.013**	-0.001
Snus																
Not using																
Former user	0.018	0.002	0.006	0.021	-0.007	**0.026**	0.007	0.006	0.004	0.001	-0.007	0.003	0.002	-0.017	-0.003	0.011
Currently user	-0.011	0.014	-0.004	**0.027**	0.001	**0.026**	0.004	**0.047**	-0.005	-0.003	**-0.022**	-0.003	**-0.057**	-0.008	0.000	0.010
Alcohol disorder test																
Not at risk																
Probably risk alcohol consumption	-0.002	**0.035**	0.016	**0.067**	-0.001	**0.030**	**0.030**	**0.060**	0.010	0.004	0.023	**0.036**	-0.061	**-0.064**	-0.007	0.019
Probably alcohol dependence	0.033	0.003	0.076	-0.003	0.001	0.052	0.113	-0.022	0.101	0.070	-0.001	0.037	-0.002	-0.084	-0.003	0.066
Availability of social interaction	0.012	**0.024**	0.005	-0.010	0.000	-0.001	-0.006	**0.028**	-0.012	0.008	-0.005	0.018	-0.003	0.009	-0.001	-0.005
Availability of attachment	-0.004	-0.016	-0.009	-0.013	0.001	-0.003	0.010	-0.014	**0.020**	-0.005	-0.005	0.016	0.006	-0.007	0.000	-0.001
Job strain																
Low strain																
Passive	-0.007	0.008	-0.007	-0.006	-0.008	0.013	0.011	0.013	0.009	**0.028**	-0.010	-0.010	**-0.041**	0.004	**-0.012**	0.008
Active	-0.010	0.024	-0.013	0.020	0.006	0.020	0.014	0.028	0.002	**0.024**	-0.012	0.022	-0.026	0.000	-0.005	**0.024**
High strain	-0.002	**0.038**	-0.013	**0.034**	-0.002	0.013	0.001	**0.036**	0.007	**0.033**	-0.019	0.021	**-0.060**	-0.002	-0.003	0.018

Bold indicates *p* < 0.05

## Abbreviations

AUDIT: Alcohol Use Disorder Identification Test
AVAT: availability of attachment
AVSI: availability of social integration
BMI: Body mass index
C: Concentration index
CVD: cardiovascular disease
CVRF: Cardiovascular risk factors
HDL: High-density lipoproteins
LDL: Low-density lipoproteins
VIP: Västerbotten Intervention Program
Yrs: years
